# Historical fragmentation and stepping‐stone gene flow led to population genetic differentiation in a coastal seabird

**DOI:** 10.1002/ece3.11204

**Published:** 2024-04-17

**Authors:** Bronwyn A. S. Harkness, Gabriela Ibarguchi, Veronica F. Poland, Vicki L. Friesen

**Affiliations:** ^1^ Department of Biology Queen's University Kingston Ontario Canada; ^2^ Present address: Environment and Climate Change Canada, National Wildlife Research Centre Ottawa Ontario Canada; ^3^ Present address: Red Deer Polytechnic Red Deer Alberta Canada; ^4^ Present address: Kambah Australian Capital Territory Australia

**Keywords:** *Cepphus columba*, conservation genetics, evolutionary genetics, introns, microsatellites, mitochondrial control region, Pigeon Guillemot, wildlife management

## Abstract

Understanding the forces that shape population genetic structure is fundamental both for understanding evolutionary trajectories and for conservation. Many factors can influence the geographic distribution of genetic variation, and the extent to which local populations differ can be especially difficult to predict in highly mobile organisms. For example, many species of seabirds are essentially panmictic, but some show strong structure. Pigeon Guillemots (*Cepphus columba*; Charadriiformes: Alcidae) breed in small colonies scattered along the North Pacific coastline and feed in shallow nearshore waters year‐round. Given their distribution, gene flow is potentially lower and population genetic structure is stronger than in most other high‐latitude Northern Hemisphere seabirds. We screened variation in the mitochondrial control region, four microsatellite loci, and two nuclear introns in 202 Pigeon Guillemots representing three of five subspecies. Mitochondrial sequences and nuclear loci both showed significant population differences, although structure was weaker for the nuclear loci. Genetic differentiation was correlated with geographic distance between sampling locations for both the mitochondrial and nuclear loci. Mitochondrial gene trees and demographic modeling both provided strong evidence for two refugial populations during the Pleistocene glaciations: one in the Aleutian Islands and one farther east and south. We conclude that historical fragmentation combined with a stepping‐stone model of gene flow led to the relatively strong population differentiation in Pigeon Guillemots compared to other high‐latitude Northern Hemisphere seabird species. Our study adds to growing evidence that Pleistocene glaciation events affected population genetic structure not only in terrestrial species but also in coastal marine animals.

## Graphical abstract

The relative influence of the microevolutionary influencing population genetic structure in highly mobile organisms is poorly understood. Here we analyze genetic variation in a coastal seabird from the northeast Pacific Ocean. We find that, unlike most high latitude, Northern Hemisphere seabirds, pigeon guillemots display significant population genetic structure, which appears to be due to a population fragmentation by Pleistocene glaciation events, and a stepping‐stone pattern of gene flow. Our study adds to growing evidence that Pleistocene glaciation events affected population genetic structure not only in terrestrial species but also in coastal marine animals.

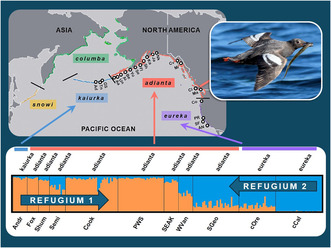

## 1 INTRODUCTION

Understanding the extent to which local populations of a species differ genetically is fundamental both to understanding of evolution and ecology and to conservation. Population genetic structure is influenced by many factors, such as dispersal patterns and population history, so can be difficult to predict in species that have not been studied directly. For example, most seabirds have strong dispersal abilities, so gene flow is generally high and population genetic structure correspondingly weak (e.g., Little Auk *Alle alle*, Wojczulanis‐Jakubas et al., 2014; reviewed in Friesen, 2015; Friesen et al., 2007; Lombal et al., 2020). However, strong population genetic structure exists in some seabird species, suggesting that gene flow is somehow restricted (e.g., Whiskered Auklet *Aethia pygmaea*, Pshenichnikova et al., 2015). In some species, genetic differentiation is explained by demographic history; for example, population fragmentation by Pleistocene glaciers has led to differentiation through genetic drift in many species (Lombal et al., 2020). In other cases, differences in nonbreeding distributions may promote population differentiation; for example, seabirds that reside at colonies year‐round or that migrate to population‐specific wintering areas may have less opportunity for gene flow than do those with a single, species‐specific migratory destination (e.g., Thick‐billed Murre *Uria lomvia*, Tigano et al., 2015, but see Quillfeldt et al., 2017). Similarly, species that forage inshore have less opportunity for gene flow among colonies than do more pelagic feeders, and accordingly, inshore foraging is sometimes associated with population genetic structure (Wiley et al., 2012). Importantly, gene flow and population genetic structure may also be influenced by the geographic distribution of colonies: species that nest in small colonies scattered along coastlines likely follow a one‐dimensional stepping‐stone model of gene flow where breeding recruits disperse only short distances from their natal colony. Under this model, gene flow is less effective at countering genetic drift than in an n‐island model (Kimura & Weiss, 1964).

Guillemots (*Cepphus* spp.; Charadriiformes: Alcidae) differ from most seabirds in several key traits, and so provide useful systems for investigating mechanisms of population differentiation in highly mobile species. Although guillemots have the potential to disperse hundreds of kilometers from natal sites (e.g., Johnston et al., 2018), several aspects of their natural history may restrict gene flow: guillemots avoid flying over large expanses of land or ice (Udvardy, 1963); they generally breed in small colonies on islands and headlands (Gaston & Jones, 1998); they forage on small demersal fish in shallow water near breeding colonies (e.g., Golet et al., 2000); and they winter close to their breeding colonies (Ewins, 2020). Furthermore, given that guillemots breed in small colonies scattered along coastlines and feed nearshore, often near sea ice (Divoky et al., 2016), they could have survived the Pleistocene glaciations in small coastal refugia (Roberts & Hamann, 2015; Waltari et al., 2007). Accordingly, Kidd and Friesen (1998a) found that mitochondrial DNA (mtDNA) sequences vary among regional populations of Black Guillemots (*C. grylle*) in the North Atlantic, due at least partly to historical fragmentation by Pleistocene glaciers.

**FIGURE 1:**
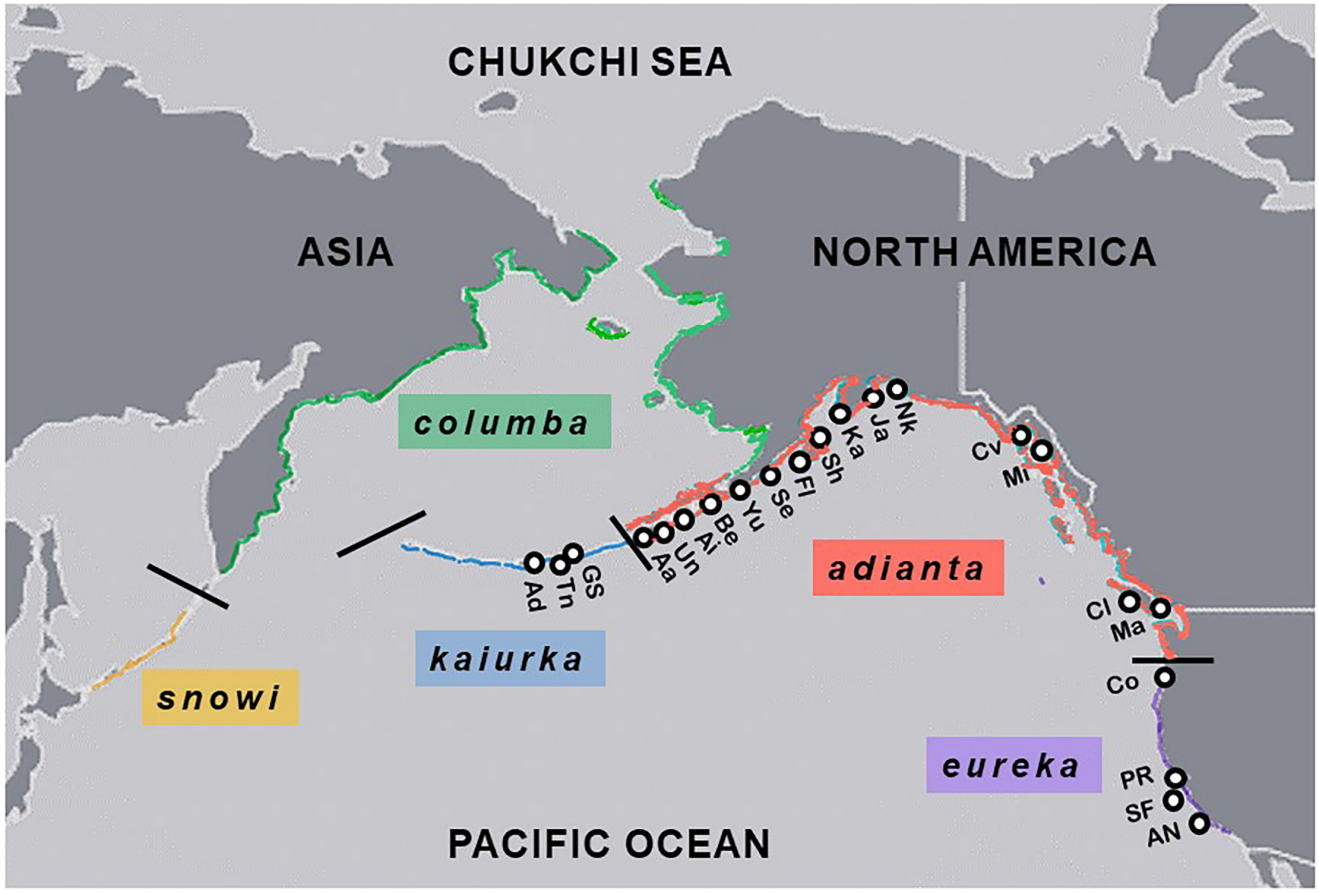
Breeding distribution (colored lines) of Pigeon Guillemots, and breeding sites where samples were collected (circles). Sampling site codes are given in Table 1. Redrawn from Udvardy (1963).

Pigeon Guillemots (*C. columba*) breed along coastlands of the North Pacific Ocean (Figure 1), with an estimated total population of less than 69,000 breeding birds (Kushlan et al., 2002). The possibility that local populations of Pigeon Guillemots may differ genetically is suggested by geographic variation in morphology: five subspecies have been designated based on clines in wing, tarsus, and culmen lengths (increasing from north to south), and quantity of white on the wing (increasing from south to north; Storer, 1952). Furthermore, several co‐distributed and ecologically similar seabird species have strong population genetic structures (e.g., Marbled Murrelets *Brachyramphus marmoratus*, Congdon et al., 2000; Kittlitz's Murrelets *B. brevirostris*, Birt et al., 2011). Kidd and Friesen (1998a) analyzed geographic variation in the conserved central domain of the mitochondrial control region (mCR) of Pigeon Guillemots and found evidence of population genetic structure. However, their geographic sampling was limited and their study included only mtDNA, which often differs in geographic variation from nuclear DNA due to differences in mode of inheritance and mutation rates (e.g., Ando et al., 2014; Eda et al., 2008; Walsh & Edwards, 2005).

In the present study, we analyzed variation in the more variable sections of the mCR and six noncoding nuclear loci among Pigeon Guillemots sampled throughout the northeastern Pacific to characterize their population genetic structure and historical demography. Given their natural history (above), we hypothesized that local populations would differ at neutral molecular loci and that genetic differences between local populations would correlate with geographic distance. Furthermore, we hypothesized that geographic variation in DNA sequences would reflect historical fragmentation driven by Pleistocene glaciation events.

## 2 METHODS

### 2.1 Sampling and DNA screening

Solid tissue or blood samples were collected from 202 Pigeon Guillemots from 22 breeding sites within 11 locations (archipelagos, inlets, and bays) ranging from the western Aleutian Islands to central California (Table 1, Figure 1). Most samples from Alaska comprised solid tissue (heart, liver, or striated muscle) from adults in breeding condition collected near colonies for dietary analyses (e.g., Hobson et al., 1994). Samples from elsewhere generally consisted of blood from adults caught at nests, opportunistically collected dead chicks and eggs, and growing coverts (“blood” or “pin” feathers) plucked from chicks (one per nest). Samples are archived at Queen's University, the Royal Ontario Museum, the Burke Museum, the American Museum of Natural History, and the University of Alaska Museum at Fairbanks. DNA was extracted using a standard protease K, RNase, phenol/chloroform technique followed by ethanol precipitation (Sambrook et al., 1989).

**TABLE 1:** Subspecies, sampling locations, location abbreviations, sampling sites, site abbreviations, and numbers (*n*) of Pigeon Guillemots analyzed for variation in mtDNA and nuclear DNA. See Figure 1 for locations of sampling sites.

Subspecies	Location	Location abbreviation	Sampling site	Site abbreviation	*n* mtDNA	*n* Nuclear
*kaiurka*	Andreanof Is.	Andr	Tanaklak I.	Tn	4	3
			Great Sitkin I.	Gs	2	1
			Adak I.	Ad	4	5
*adianta*	Fox Is.	Fox	Anangula I.	Aa	1	1
			Unalaska I.	U n	2	2
			Aiktak I.	Ai	5	4
	Shumigan Is.	Shum	Belkofski Bay	Be	3	3
			Yukon Hr.	Yu	4	4
	Semidi Is.	Semi	Semidi Is.	Se	4	5
			Flat I.	Fl	3	5
	Cook Inlet	Cook	Shuyak I.	Sh	2	3
			Kachemak Bay	Ka	32	30
	Prince William Sound	PWS	Jackpot I.	Ja	12	12
			Naked I.	Nk	18	17
	Southeast Alaska	SEAK	Midway I.	Mi	6	6
			Couverden I.	Cv	3	3
	West Vancouver I.	WVan	Cleland I.	Cl	7	9
	Strait of Georgia	SGeo	Mandarte I.	Ma	29	29
*eureka*	Central Oregon	cOre	Coos Bay	Co	24	23
	Central California	cCal	Point Reyes National Park	PR	1	1
			Southeast Farallon	SF	34	28
			Año Nuevo I.	AN	2	0
Total					202	194

Guillemot‐specific DNA primers (CGL56 and CGH549, and CGL486 and CGH1006) were used to amplify and sequence two overlapping fragments of the mCR, including parts of Domains I, II, and III, following protocols detailed in Kidd and Friesen (1998b). Sequences were obtained for 723 base pairs (bp) of the mCR, excluding the first 141 bp downstream (3′) of primer CgL56 and a 92 bp region between primers CgL486 and CgH589, both of which were ambiguous due to highly repetitive simple sequence motifs. Sequences were aligned using the program BioEdit (Hall, 1999), and haplotypes were identified using the program TCS (v 1.13, Clement et al., 2000).

The presence of length variation was determined for four microsatellite loci (Appendix 1; Table A1) either by electrophoresis through polyacrylamide gels (Ibarguchi et al., 2000) or using the GenomeLab GeXP Genetic Analysis System (Beckman Coulter Inc., USA) and associated software (10.2.3). Multiple samples were analyzed using both systems to test both for repeatability and for scoring differences due to platform effects; none were found.

Variation in six nuclear introns (Appendix 1; Table A1) was screened using a combination of single‐stranded conformational polymorphisms (SSCPs) and direct sequencing as in Friesen et al. (1997). Individuals from southeast Alaska were sequenced directly using a 3730xl DNA Analyzer Platform (Applied Biosystems, CA) operated by Genome Quebec (McGill University, Montreal, Quebec). Sequences were trimmed and aligned using the program Geneious (Kearse et al., 2012), variable sites were scored from chromatograms, and haplotypes were phased using the program PHASE (v. 2.1; Stephens & Donnelly, 2003; Stephens et al., 2001).

### 2.2 Tests of assumptions and variability

The program MICROCHECKER (van Oosterhout et al., 2004) was used to check microsatellites for null alleles. Haplotype frequencies, haplotype diversity (*h*, Nei, 1987), and nucleotide diversity (π, Tajima, 1983) were calculated for mCR sequences, and allele frequencies and heterozygosities were calculated for nuclear loci for each sampling location using ARLEQUIN (v. 3.5, Excoffier & Lischer, 2010). ARLEQUIN was also used to test mitochondrial variation for deviations from neutrality using Ewens‐Watterson and Chakraborty tests (Chakraborty, 1990; Ewens, 1972; Watterson, 1978) and to test nuclear loci for deviations from Hardy–Weinberg proportions and linkage equilibrium.

### 2.3 Population genetic structure

The proportion of genetic variation distributed among populations (Φ_ST_ for mCR sequences; *F*_ST_ for nuclear loci) was calculated by analysis of molecular variance (AMOVA) in ARLEQUIN, both for the entire dataset and for pairwise comparisons of sampling locations. Statistical significance was assessed by randomization using 10,000 permutations of the data with a rejection level (*α*) of .05. Kimura's 2‐parameter model of sequence evolution (Kimura, 1980) with a gamma value of .42 was used for mCR sequences (Marshall & Baker, 1997); use of the Tamura–Nei model (Tamura & Nei, 1993) as in Ni et al. (2012) gave virtually identical results. Potential Type I statistical errors were addressed by applying Benjamini–Yekutieli (B–Y) corrections to pairwise comparisons in R (Benjamini & Yekutieli, 2001; Narum, 2006; R Core Team, 2018).

A principal component analysis (PCA) was performed on the nuclear data in R (3.3.1, R Core Team, 2017) using the packages *adegenet* (Jombart, 2008) and *ade4* (Dray & Dufour, 2007). PCAs were run with samples grouped either by sampling location or by subspecies.

The program STRUCTURE 2.3.4 (Pritchard et al., 2000, 2010) was used as an additional test of population structure in nuclear variation. The program was initially run under the admixture model with correlated allele frequencies and without sampling location as prior information, with a burn‐in of 10,000 iterations and 100,000 iterations after the burn‐in. Following the recommendation of Hubisz et al. (2009) for species with weak structure, the model was subsequently run without admixture, with sampling location as prior information. Each value of *K* (number of genetic populations) from 1 to 5 was run 10 times, and delta *K* (Δ*K*, the most probable number of genetic populations) was calculated using STRUCTURE HARVESTER (Earl & vonHoldt, 2012; Evanno et al., 2005). Because Δ*K* cannot test if the most likely value of *K* is 1, the log‐likelihood of each *K* value (ln Pr(*x*|K)) also was calculated using the equation provided in the STRUCTURE manual (Pritchard et al., 2010). Membership probabilities were averaged among runs using CLUMPP (v.1.1.2, Jakobsson & Rosenberg, 2007) and results were displayed using DISTRUCT (v.1.1, Rosenberg, 2004).

Because the program STRUCTURE may detect artificial genetic clusters under isolation by distance (Perez et al., 2018), Mantel tests (Mantel, 1967) were used to test for correlations between pairwise estimates of Slatkin's linearized Φ_ST_ (mCR) or *F*_ST_ (mCR and nuclear loci) with log‐transformed geographic distance between sampling locations using the R package *ecodist* (Goslee & Urban, 2007) with 10,000 permutations of the data. The shortest distance between sampling locations was calculated using the package ‘*geosphere*’ (Hijmans et al., 2017). Where samples were collected from multiple breeding sites within a location (e.g., for comparisons within Andreanof Islands, Table 1), the geographic midpoint of sampling sites was used.

### 2.4 Demographic history

#### 2.4.1 Mitochondrial gene trees

Historical fragmentation should leave signatures of divergence in mitochondrial gene sequences. To test for population fragmentation by Pleistocene glaciers, a statistical parsimony network was derived for mCR sequences using PopART (Clement et al., 2002; http://popart.otago.ac.nz). Nucleotide sites with undefined states were masked. The program BEAST 1.8.4 was also used to construct a gene tree for the mCR sequences (Drummond et al., 2012). Individuals were grouped by sampling location (Table 1), and the gene tree was rooted with sequences from one Spectacled Guillemot (*C. carbo*) and one sample of each of four subspecies of Black Guillemot (*C. g. arcticus*, *C. g. grylle*, *C. g. mandti*, and *C. g. ultimus*; GenBank Accession Numbers AF027220, AF027225, AF027231, AF027233, and AF027251; Kidd & Friesen, 1998b). The Hasegawa‐Kishino‐Yano substitution model (HKY) with a proportion of invariable nucleotide sites (*I* = 0.796) and gamma distribution (*G* = 0.733; Hasegawa et al., 1985) was selected as the best model using the Bayesian information criterion (BIC) in jModelTest2 (Darriba et al., 2012). A strict molecular clock with a mutation rate of 7.4%/my (million years; Wenink et al., 1996) and the coalescent tree prior that assumes a constant population size with Jeffrey's prior (Drummond et al., 2005; Jeffreys, 1946; Kingman, 1982) were selected. The BEAST analysis was run for 10^7^ MCMC (Markov chain Monte Carlo) runs with a burn‐in of 10^6^ runs. TRACER 1.6 (Rambaut et al., 2014) was used to analyze the trace files generated by BEAST and to assess convergence. The analysis was run 3 times and output files were combined using LogCombiner (v. 1.8.4), with a burn‐in of 10% of trees for each original file. A consensus tree was generated in TreeAnnotator (v. 1.8.4, Drummond et al., 2012) and the tree was displayed graphically using FigTree (v. 1.4.3, Rambaut, 2007).

#### 2.4.2 Population simulations

**FIGURE 2:**
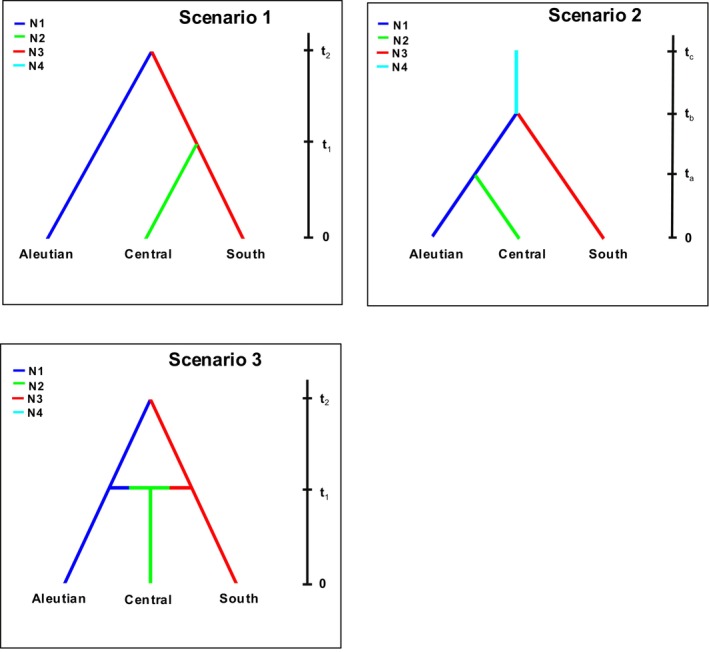
Alternative demographic histories tested using DIYABC. All three scenarios assume three population groups: (1) “Aleutian Islands” (Andreanof Is., and Fox Is.); (2) “Central” (Shumigan Is., Semidi Is., Cook Inlet, Prince William Sound, and Southeast Alaska); and (3) “South”: West Vancouver Is., Strait of Georgia, Central Oregon, and Central California at the present time (*t*0), and these populations diverged in the past. The time scale is shown on the right.

To test alternative models of population history, mCR variation was compared to simulated variation generated under three potential scenarios using coalescent‐based approximate Bayesian computation (ABC) in DIYABC (Cornuet et al., 2008). This approach involves modeling different historical scenarios and simulating population sequence evolution to fit each scenario. The simulated datasets are then compared to indices of variation in the observed dataset to determine which scenario best fits the data. Based on the observed geographic distribution of mCR variation (see Section 3), samples were grouped into three regions to test alternative roles of Pleistocene refugia: “Aleutians” (Andreanof Is. and Fox Is.); “Central” (Shumigan Is., Semidi Is., Cook Inlet, Prince William Sound, and Southeast Alaska); and “South” (West Vancouver Is., Strait of Georgia, Central Oregon, and Central California). Scenario 1 (Figure 2) simulated two refugial populations during the last glaciation, with Aleutian populations diverging from the others pre‐glaciation (*t*2) with no subsequent gene flow, and central and southern populations diverging post‐glaciation (*t*1). Scenario 2 simulated isolation of Pigeon Guillemots in a single southern glacial refugium, followed by step‐wise colonization northward post‐glaciation. Scenario 3 tested that Pigeon Guillemots were historically isolated in two refugia (Aleutian and southern) at *t*2 with secondary contact in the central region post‐glaciation at *t*1. The prior distribution for all historical parameters was set to uniform. Due to the absence of published data for genetically effective population size (*N*_e_), broad priors were used (10–100,000). Mean generation time of Pigeon Guillemots was estimated to be 8.8 years (Hudson, 1985). Times of divergence of the ancestral populations (*t*2 and *tc*) were set to 1250–100,000 generations ago (11,000–880,000 years ago [ya]; i.e., before the end of the Wisconsin glaciation). Admixture events and more recent divergences (*t1*, *ta*, and *tb*) were set to 0–1250 generations ago (present day – 11,000 ya; i.e., after the end of the Wisconsin glaciation). Rate of admixture (*ra*) was left as the default (0.001–0.999), and the HKY + I + G substitution model was selected, as above. Mean mutation rate was set to 6.5 × 10^−7^ per site per generation based on a sequence divergence rate of 7.4%/my and a generation time of 8.8 years (above). Eight single‐sample summary statistics were used for each population: number of distinct haplotypes, number of segregating sites, mean pairwise difference, variance of the number of pairwise differences, Tajima's *D* statistics (Tajima, 1989), number of private segregating sites, mean of the numbers of the rarest nucleotide at segregating sites, and variance of the numbers of the rarest nucleotide at segregating sites. Additionally, 5 two‐sample summary statistics were used: number of distinct haplotypes in the pooled sample, number of segregating sites in the pooled sample, mean of within‐sample pairwise differences, mean of between sample pairwise differences, and pairwise *F*_ST_ (Hudson et al., 1992). Three million simulations were run for each scenario, and posterior probabilities were compared using logistic regression (Cornuet et al., 2010; Inoue et al., 2014). Type I and Type II error rates were estimated using the method described by Cornuet et al. (2010) and Inoue et al. (2014).

## 3 RESULTS

### 3.1 Molecular variation and tests of assumptions

Mitochondrial control region sequences were similar to those published previously for guillemots (Kidd & Friesen, 1998a, 1998b), and contained the conserved sequence blocks typical of other avian species (F, D, and C Boxes and CSB‐1; Marshall & Baker, 1997). All mitochondrial and intron sequences have been deposited in GenBank, accession numbers PP593249‐PP593336. Population‐specific haplotype and allele frequencies have been deposited in Dryad https://doi.org/10.5061/dryad.m37pvmd9m. Eighty‐five mCR haplotypes, defined by 78 variable sites, were identified. Fifty‐nine variable nucleotide sites occurred in Domain I, 17 in Domain II, and two in Domain III. Ewens‐Watterson and Chakraborty tests did not detect any deviations from neutrality except in the Strait of Georgia (Mandarte Island): in the Chakraborty test, the Strait of Georgia samples had significantly more haplotypes than expected (14 observed vs. 8.6 expected, *p* = .02; Table 2); however, these samples did not show a significant deviation from neutrality using the Ewens‐Watterson test.

**TABLE 2:** Number of haplotypes, haplotype diversity (*h*), nucleotide diversity (*π*) as a percent (+1 standard deviation), observed/expected *F* values for Ewens‐Watterson test of neutrality, and probabilities of neutrality from Chakraborty's test; one significant value is in bold.

Sampling location	Number of haplotypes	*h*	*π*	Ewens‐Watterson	Chakraborty
Andr	10	0.98 ± 0.05	0.73 ± 0.44	0.12/0.12	0.76
Fox	6	0.89 ± 0.11	1.25 ± 0.73	0.22/0.20	0.56
Shum	7	1.00 ± 0.08	1.19 ± 0.72	NA	NA
Semi	7	1.00 ± 0.08	0.79 ± 0.49	NA	NA
Cook	21	0.96 ± 0.02	1.04 ± 0.55	0.07/0.07	0.65
PWS	16	0.95 ± 0.02	0.68 ± 0.38	0.08/0.10	0.87
SEAK	8	0.97 ± 0.06	0.47 ± 0.30	0.14/0.14	0.77
WVan	3	0.76 ± 0.11	0.29 ± 0.21	0.35/0.45	0.86
SGeo	14	0.83 ± 0.07	0.51 ± 0.29	0.20/0.12	**0.02**
cOre	10	0.90 ± 0.04	0.58 ± 0.33	0.14/0.17	0.78
cCal	12	0.88 ± 0.03	0.47 ± 0.27	0.14/0.16	0.66

*Note*: ‘NA’, not applicable due to small sample size. Sampling location abbreviations are in Table 1.

All nuclear loci were variable. No null alleles were detected by MICROCHECKER for microsatellite data. None of the nuclear loci showed significant deviations from Hardy–Weinberg proportions within any of the sampling locations except for Ribosomal Protein 40 (RP4 intron V) in the Strait of Georgia (Table 3). Six of the 11 sampling locations showed evidence of linkage disequilibrium between different loci (Table A2). Tests for population genetic structure were rerun excluding one locus from each pair and results remained consistent; all loci were therefore retained for subsequent analyses.

**TABLE 3:** Observed/expected heterozygosity estimates for nuclear loci screened for Pigeon Guillemots. Sampling location abbreviations are in Table 1.

Sampling location	Microsatellites				Introns	
	Dpu16	Uaa5‐8	Cco5‐9	Cco5‐21	Cytochrome *c* intron I	Ribosomal protein 40 intron V
Andr	NA	0.89/0.75	0.67/0.68	0.86/0.86	0.89/0.74	0.44/0.63
Fox	NA	0.43/0.49	1.00/0.85	0.85/0.97	0.86/0.63	0.29/0.26
Shum	NA	0.57/0.65	0.86/0.85	0.86/0.78	0.71/0.54	0.14/0.14
Semi	0.2/0.19	0.40/0.57	0.9/0.82	0.80/0.79	0.40/0.57	0.40/0.35
Cook	0.03/0.03	0.64/0.59	0.85/0.85	0.79/0.78	0.67/0.62	0.30/0.40
PWS	NA	0.59/0.59	0.86/0.84	0.79/0.80	0.59/0.58	0.38/0.31
SEAK	NA	0.67/0.50	0.78/0.71	0.56/0.55	0.44/0.57	0.22/0.40
WVan	0.13/0.13	0.33/0.57	0.88/0.74	0.67/0.56	0.63/0.58	0.50/0.50
SGeo	NA	0.49/0.56	0.72/0.68	0.45/0.46	0.62/0.60	0.17/0.36*
cOre	0.26/0.23	0.45/0.63	0.52/0.50	0.74/0.71	0.61/0.49	0.43/0.39
cCal	0.12/0.10	0.69/0.65	NA	0.66/0.61	0.21/0.25	0.17/0.23

* Significantly different at *α* = .05 both before and after B–Y correction.

Six alleles, defined by variation at four (Cytochrome *C* intron I) and 15 nucleotide sites (RP40) were found within each of the two introns. Alleles differed by one to 12 substitutions. Number of alleles at microsatellite loci ranged from 3 to 11. For all six nuclear loci, several alleles were present at high frequency in most sampling locations, and the remaining alleles occurred in only one or two individuals each.

### 3.2 Population genetic structure

Of the 81 mCR haplotypes, 64 were private (found in only one sampling location), including five at high frequencies (over 25%). With four exceptions, shared haplotypes were found in geographically adjacent locations; the four that were found in non‐adjacent locations occurred at low frequency in one location. The global AMOVA indicated statistically significant geographic structure in sequence variation (Φ_st_ = 0.39, *p* < .001), and pairwise estimates of Φ_st_ were significant for most comparisons (Table 4).

**TABLE 4:** Estimates of *F*_ST_ based on six nuclear loci (above diagonal) and estimates of Φ_ST_ based on sequence variation in the mitochondrial control region (below diagonal) for pairwise comparisons of sampling locations. Location abbreviations are in Table 1.

	Andr	Fox	Shum	Semi	Cook	PWS	SEAK	WVan	SGeo	cOre	cCal
Andr		0.01	0.04	0.04	0.02	0.03*	0.11**	0.08*	0.14**	0.11**	0.26**
Fox	0.11*		0.01	0.01	0.02	0.00	0.07**	0.07*	0.13**	0.10**	0.26**
Shum	0.48**	0.39**		0.00	0.03*	0.01	0.11**	0.08*	0.08**	0.08**	0.21**
Semi	0.57**	0.47**	−0.01		0.01	−0.01	0.11**	0.01	0.05*	0.05**	0.16**
Cook	0.50**	0.49**	0.14*	0.01		0.00	0.11**	0.04*	0.10**	0.09**	0.18**
PWS	0.60**	0.55**	0.17**	−0.01	0.04*		0.11**	0.05**	0.10**	0.09**	0.18**
SEAK	0.66**	0.58**	0.14**	0.13*	0.15**	0.21**		0.06*	0.12**	0.12**	0.26**
WVan	0.67**	0.58**	0.12*	0.13*	0.13*	0.12*	0.27**		0.01	0.03*	0.18**
SGeo	0.66**	0.62**	0.15**	0.11*	0.17**	0.15**	0.13**	0.00		0.07**	0.16**
cOre	0.66**	0.61**	0.21**	0.24**	0.28**	0.29**	0.27**	0.19*	0.21**		0.02*
cCal	0.73**	0.70**	0.38**	0.44**	0.42**	0.47**	0.46**	0.45**	0.45**	0.11**	

* Significantly different from 0 at *α* = .05 before B–Y correction.

** Significantly different from 0 at *α* = .05 after B–Y correction.

Estimates of global population genetic structure for nuclear loci were statistically significant but slightly lower than for the mCR (*F*_ST_ = 0.09 for all nuclear loci; *F*_ST_ = 0.11 for microsatellites only; *F*_ST_ = 0.05 for introns only; all *p* < .001). Pairwise estimates of *F*_ST_ were statistically significant for most location comparisons for all nuclear loci combined and for microsatellites and for some pairwise comparisons for introns (Table 4; Table A3).

**FIGURE 3:**
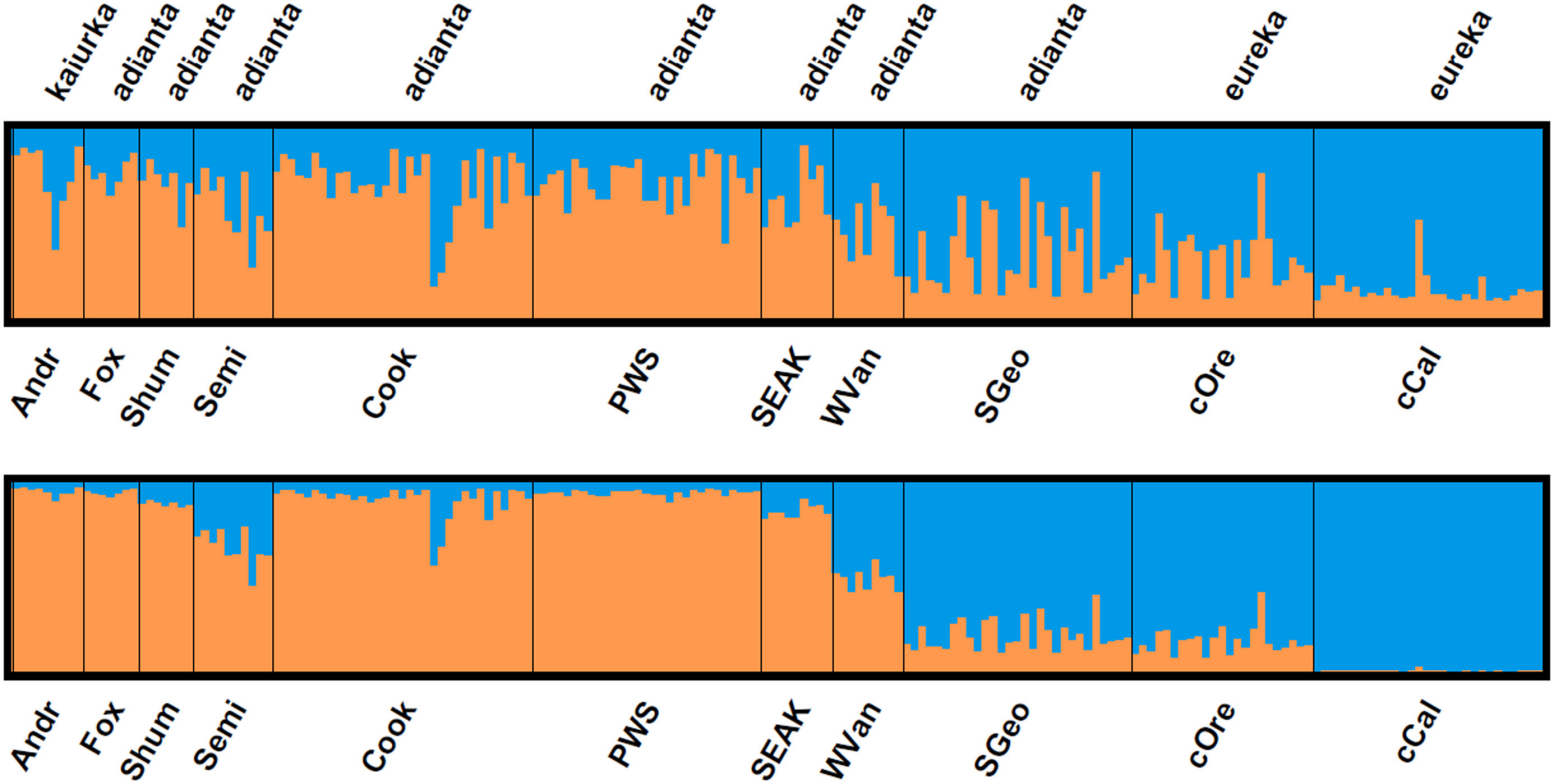
Results of principal component analysis of nuclear variation in Pigeon Guillemots, with samples grouped by (a) population, and (b) subspecies. (a) 1 = Andreanof Is., 2 = Fox Is., 3 = Shumigan Is., 4 = Eastern Alaska Peninsula, 5 = Cook Inlet, 6 = Prince William Sound, 7 = South East Alaska, 8 = West Vancouver Is., 9 = Strait of Georgia, 10 = Central Oregon, 11 = Central California. (b) 1 = *kaiurka* (Andreanof Is.), 2 = *adianta* (Shumigan Is., Eastern Alaska Peninsula, Cook Inlet, Prince William Sound, South East Alaska, West Vancouver Is., Strait of Georgia), 3 = *eureka* (Central Oregon, Central California).

No population genetic structure was obvious in the PCA when Pigeon Guillemots were grouped by sampling location (Figure 3a). When populations were grouped by subspecies, *C. kaiurka* and *C. eureka* were largely separated from each other, whereas *C. adianata* overlapped with both other subspecies (Figure 3b), with the first three principal components explaining 17.5% of the variance in both analyses.

**FIGURE 4:**
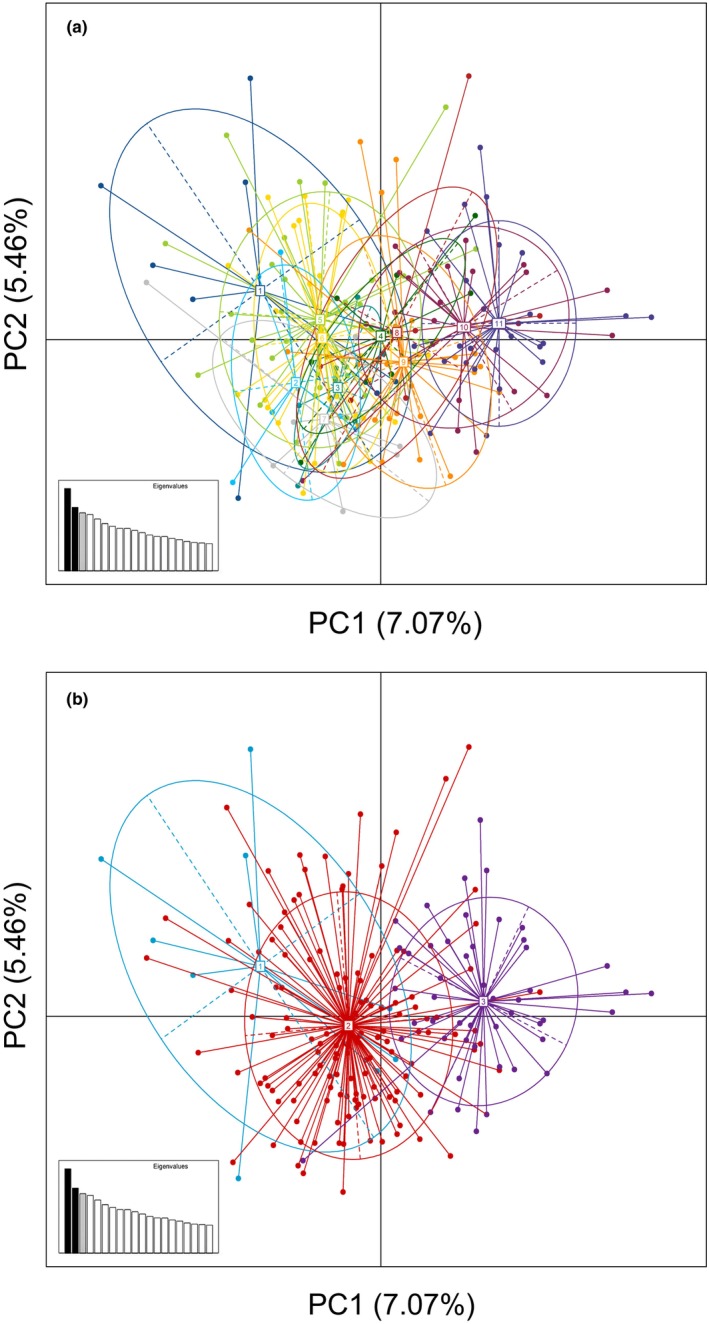
Probabilities of assignment of individuals to each of two genetic populations (*K* = 2) derived from the program STRUCTURE, based on genetic variation in nuclear loci of Pigeon Guillemots. Top: results for runs allowing admixture, not using sampling location as prior information; bottom: results for runs without admixture, using sampling location as prior information. Populations are ordered as they appear geographically, from northwest to southeast (left to right). Location abbreviations as in Table 1.

Estimates of both Δ*K* and Ln Pr(*x*|*K*) from analyses of nuclear variation using STRUCTURE indicated that the most likely value of *K* for Pigeon Guillemots is 2 (Figure 4; Table A4). Genetic groups were not completely segregated geographically, with most individuals showing some probability of assignment to both genetic populations.

**FIGURE 5:**
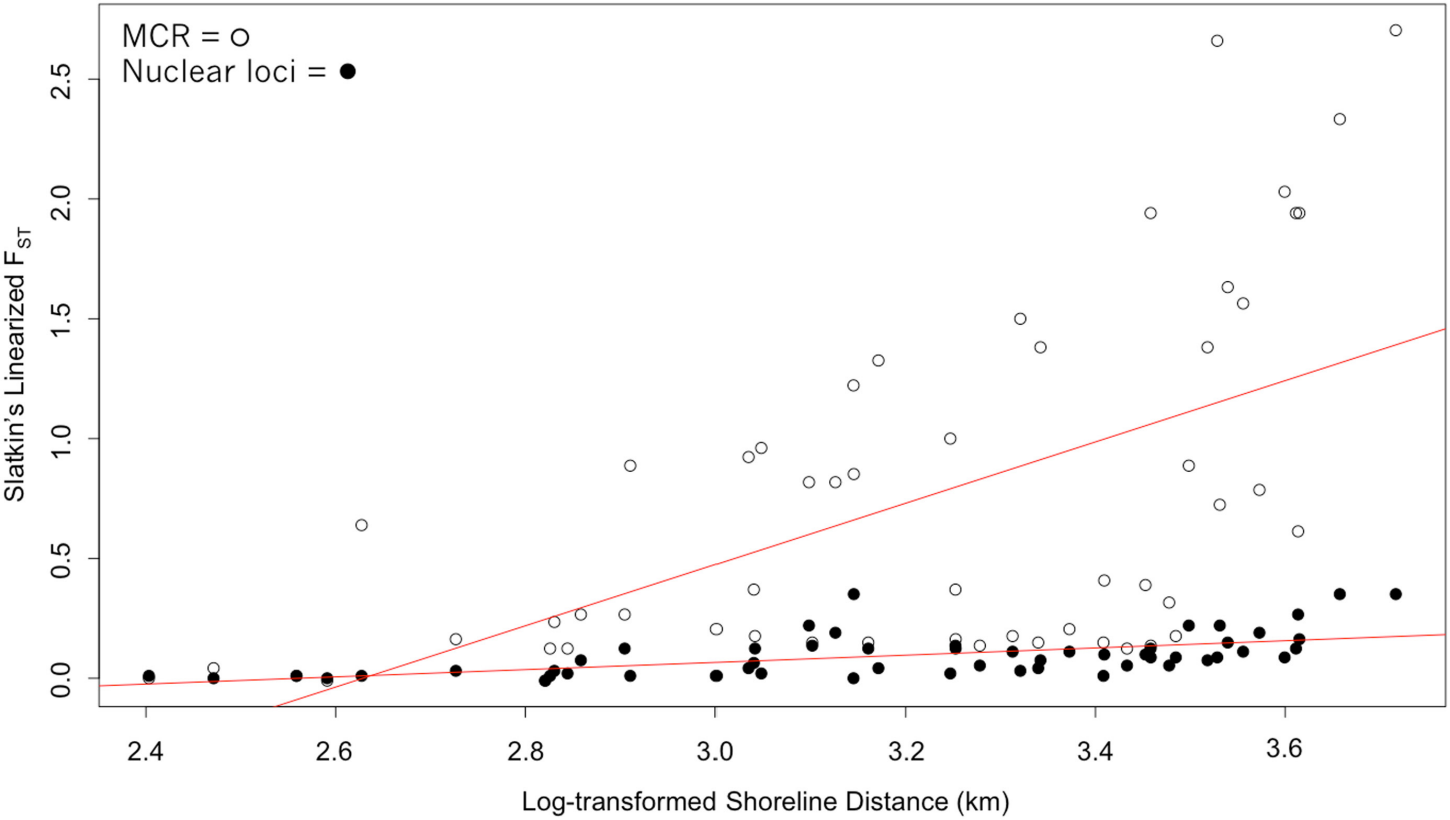
Genetic distance (Slatkin's linearized *F*_ST_) versus geographic distance (log‐transformed linear distance) between sampling locations for Pigeon Guillemots. White circles represent comparisons made using mitochondrial data and black circles represent comparison made using nuclear data. The lines of best fit for both the analysis with mitochondrial data and with nuclear data are shown in red.

**FIGURE 6:**
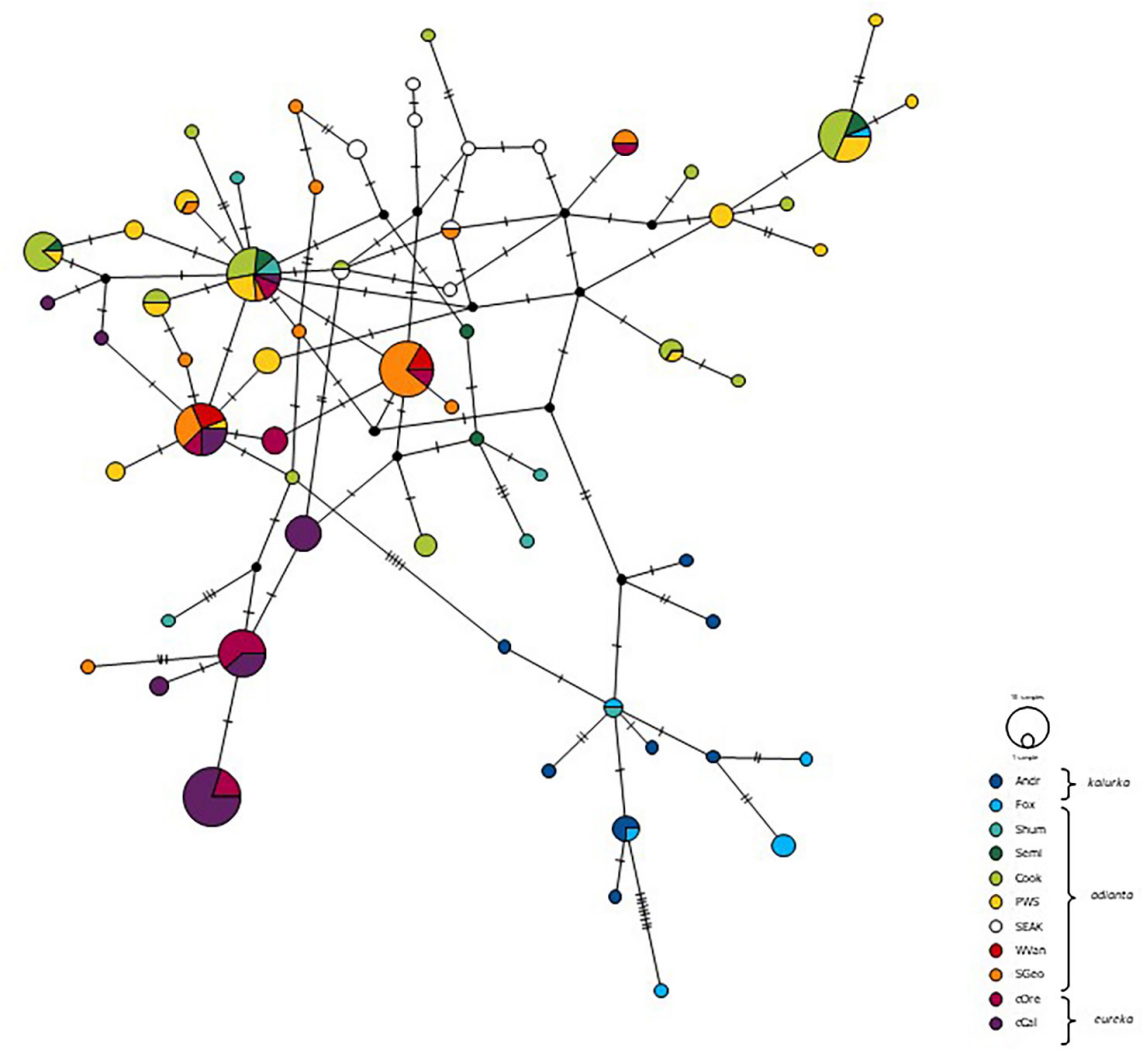
Statistical parsimony haplotype network derived for control region sequences of Pigeon Guillemots using PopART. Sizes of circles are proportional to haplotype frequencies. Black dots represent inferred intermediate haplotypes that were not found in the present sampling. Haplotype names have been removed for clarity. Region abbreviations as in Table 1.

**FIGURE A1:**
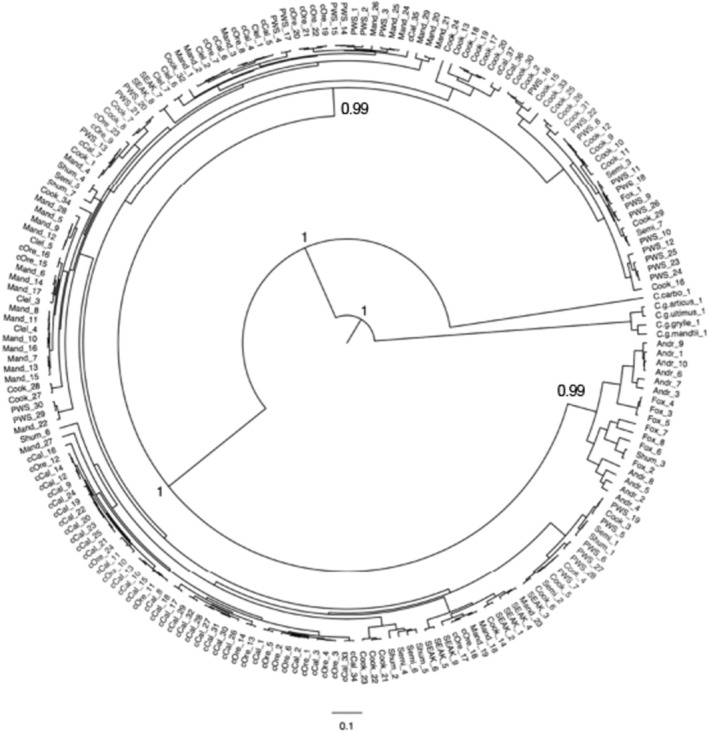
Phylogenic relationships among mitochondrial control region sequences of Pigeon Guillemots from different sampling location, generated using BEAST. *C. grylle* and *C. carbo* are used as an outgroup. Posterior probabilities are shown at major nodes. Nodes with low posterior probabilities are not labeled.

Mantel tests for correlations between genetic and geographic distance were statistically significant both for mCR variation (*r* = .56, *p* < .05) and for all nuclear loci (*r* = .59, *p* < .05; Figure 5). Given the divergence between mtDNA haplotypes of samples from the Aleutian Islands versus elsewhere, Mantel tests were repeated after removing samples from the Aleutian Islands (Andreanof Island and Fox Island), as most of these samples were distinct from those from other locations (Figure 6, Figure A1): correlations remained significant but slightly weaker (mCR, *r* = .52, *p* < .05; all nuclear loci, *r* = .52, *p* < .05). The Mantel test for mtDNA variation was also rerun based on haplotype frequencies only (i.e., *F*_ST_ rather than ϕ_ST_): the result was no longer significant (*r* = .14, *p* = .18; plot not shown), suggesting that the correlation is driven at least partially by divergence between Aleutian and other mtDNA lineages.

### 3.3 Demographic history

Divergence times for the mtDNA sequences of the Black Guillemot outgroup versus Pacific species of guillemots were estimated from BEAST at between 0.63 and 1.2 mya (with a posterior probability of 1), which is slightly more recent than the estimate of Kidd and Friesen (1998a; 1.5 mya; Figure A1). Spectacled and Pigeon Guillemot mtDNA lineages were estimated to have diverged between 0.43–0.87 mya (with a posterior probability of 1), which is similar to the date estimated by Kidd and Friesen (1998a; 0.8 mya).

Both the TCS and BEAST gene trees separated mtDNA sequences of most Pigeon Guillemots from the Aleutian Islands (Andreanof and Fox Islands) from those from other sampling locations, with two exceptions: sequence from one individual from Shumigan Island grouped with the Aleutian samples, and sequence from one individual from Fox Island mixed with samples from the other locations (Figure 6 and Figure A1); these haplotypes were confirmed both by analysis of SSCPs and by direct sequencing. Support from BEAST for monophyly of the mtDNA lineages of Pigeon Guillemots from the Aleutian Islands versus those from elsewhere was strong, with posterior probabilities of 1. The mtDNA lineages of Pigeon Guillemots in the Aleutian Islands versus elsewhere were estimated to have diverged between 0.16–0.32 mya. Relationships among mtDNA lineages within these two clades were not well resolved.

Scenario 1 (isolation of guillemots in two refugial populations, followed by divergence within the southern population) was the most highly supported by DIYABC, with a strong posterior probability (0.83). The other two scenarios had much lower support. Type I and Type II error rates for all three scenarios were low (Table A5).

## 4 DISCUSSION

Our objectives were to characterize population structure in neutral molecular loci in a coastally distributed seabird and to test for historical fragmentation by Pleistocene glaciers. We found that mCR sequence variation was strongly structured geographically and that significant population structure also occurred in nuclear DNA despite the small number of loci. Genetic differences between sampling locations correlated with geographic distance, supporting a stepping‐stone model of gene flow. Evidence was also found for population fragmentation by Pleistocene glaciation events.

### 4.1 Population genetic structure

Estimates of ΦST for mCR sequences were significantly greater than zero between almost all sampling locations, with many private haplotypes and significant divergence between most haplotypes sampled from the Aleutian Islands versus elsewhere. Nuclear variation also was structured geographically: estimates of *F*_ST_ between many sampling locations were statistically significant; PCA indicated some differentiation among subspecies; and analyses with STRUCTURE indicated two genetic populations. Although geographic structuring in nuclear loci was weaker than in mtDNA, this pattern is common in birds (e.g., Sonsthagen et al., 2011; Zink & Barrowclough, 2008) and consistent with the lower effective population size and correspondingly stronger genetic drift in mtDNA compared to nuclear DNA. Furthermore, a pattern of isolation by distance was suggested by several results: haplotype sharing between neighboring locations; correlations between genetic and geographic distance for both mCR and nuclear variation; and mixed probabilities of assignment of individuals to each of two genetic populations by STRUCTURE, with assignment probabilities changing roughly clinally. Additionally, the existence of four mitochondrial haplotypes shared between non‐adjacent sampling locations is suggestive of occasional long‐distance gene flow.

### 4.2 Demographic history

In theory, the extent of population genetic differentiation is influenced by many factors, one of which is the pattern of gene flow (Wright, 1969). As described in the Introduction, guillemots likely disperse according to a one‐dimensional stepping‐stone model of gene flow (along coastal habitat). If migrants follow an n‐island model of gene flow (i.e., are exchanged at random among breeding locations), then in theory one migrant per generation will homogenize allele frequencies, and genetic differentiation between locations will be independent of geographic distance (Wright, 1969). However, if migrants disperse according to a stepping‐stone model (i.e. primarily between neighboring breeding locations), then gene flow will be less effective at counteracting genetic drift (Kimura & Weiss, 1964), and genetic differentiation between locations should increase with distance (Hutchison & Templeton, 1999; Mantel, 1967). Genetic distance correlated with geographic distance in several seabirds reviewed by Friesen et al. (2007) and a variety of other taxa (reviewed in Perez et al., 2018; e.g., the cup coral *Balanophyllia elegans*, Hellberg, 1995; Red Drum *Sciaenops ocellatus*, Gold et al., 2001; Arizona Tree Frog *Hyla wrightorum*, Mims et al., 2016; Eurasian Badger *Meles meles*, Pope et al., 2006).

Correlations between genetic and geographic distance can result from either recent differentiation in situ or historical fragmentation followed by population expansion and secondary contact (Hutchison & Templeton, 1999). In the present study, both of the mitochondrial gene trees showed a disjunction in sequence variation between most haplotypes from the Aleutian Islands and locations farther east and south. The distinctiveness of the Aleutian haplotypes suggests that guillemots from these islands were historically isolated from those elsewhere. Estimates of Φst and *F*ST were also greatest between samples from the Aleutian Islands and those elsewhere, suggesting that they experienced limited gene flow in more recent years. The split between mtDNA lineages of the Aleutian Islands and other populations was estimated by BEAST to have occurred 0.16–0.36 mya, before or during the Illinoian glaciation when ice sheets would have fragmented the Pacific coast of North America (Dyke, 2004). Results from DIYABC using mtDNA also suggested that, of the three demographic histories modeled, the most likely scenario was a historical split between populations in the Aleutian Island and those farther east and south prior to the Wisconsin glaciation, with the latter populations diverging from each other post‐glaciation. Conclusions drawn from DIYABC should be treated with caution, since the program can only test models that are supplied by the researcher, and results here are based on only mtDNA variation. Nonetheless, our results suggest that differentiation of Aleutian Island vs. other North American Pigeon Guillemots may be explained at least in part by historical fragmentation, probably by extensive Pleistocene ice fields that would have separated tracts of rocky coastline from each other. These findings are consistent with Udvardy's (1963) hypothesis that geographic variation in Pigeon Guillemots is due to isolation in multiple glacial refugia.

Pleistocene refugia are often discussed from the perspective of terrestrial species that cannot travel over large expanses of ice (e.g., Holder et al., 2000; Wang et al., 2021). Coastal species such as guillemots could potentially have persisted in small numbers in ice‐free outcrops (e.g., Haida Gwaii). However, the Cordilleran Ice Sheet at its peak extended to the edge of the continental shelf, potentially fragmenting even coastal marine species between the western end of the Aleutian Islands and northern Washington (Dyke, 2004). Many other coastal species exhibit population differentiation potentially associated with historical fragmentation by Pleistocene glaciers in the North Pacific (Dyke & Prest, 1987; e.g., Canada Goose *Branta canadensis*, Pierson et al., 2000; Marbled Murrelet, Congdon et al., 2000; Common Eider *Somateria mollissima*, Sonsthagen et al., 2011; Steller's Sea Lion *Eumetopias jubatus*, Hoffman et al., 2006; Sea Otter *Enhydra lutris*, Larson et al., 2012), indicating a common influence of Pleistocene glaciers even on non‐terrestrial species.

### 4.3 Taxonomic and conservation implications

According to Storer (1952), subspecies of Pigeon Guillemots show marked morphological variation along a latitudinal cline. While genetic variation in this species varies geographically, population differences at neutral molecular loci do not fully align with current subspecies delineations: samples of *C. c. adianata* from the Fox Islands grouped with *C. c. kaiurka* on the mitochondrial gene trees; furthermore, demographic simulations suggested that guillemots from the Aleutian Islands survived the Pleistocene in a refugium separate from guillemots farther east and south. Thus, the location of the geographic boundary between these two subspecies should be revisited. Outside the Aleutian Islands, variation in neutral loci tended to increase with geographic distance between sampling locations, blurring the lines between *C. c. adianta* and *C. c. eureka*. Perez et al. (2018) cautioned against inferring population units from clustering programs such as STRUCTURE under patterns of isolation by distance. Analysis of a larger number of loci may improve resolution of fine population structure and subspecies boundaries. Samples of *C. c. columba* and *C. c. snowi* also need to be included in future analyses.

Identification of subspecies or other distinct lineages is fundamental for informing management and in conservation planning. For species with strong genetic structuring, natural and anthropogenic events that affect particular populations (or colonies, such as in Pigeon Guillemots) can potentially impact the species in a disproportionate way if they lead to loss of unique genetic diversity and/or local adaptations. Prior knowledge about the ecology and evolutionary trajectories of species is highly beneficial also for monitoring and to aid recovery and restoration efforts following larger‐scale disturbance events such as oil spills.

## AUTHOR CONTRIBUTIONS

**Bronwyn A. S. Harkness:** Conceptualization (supporting); data curation (supporting); formal analysis (lead); funding acquisition (supporting); investigation (supporting); methodology (lead); project administration (supporting); validation (lead); visualization (lead); writing – original draft (lead); writing – review and editing (supporting). **Veronica F. Poland:** Data curation (supporting); formal analysis (supporting); methodology (supporting); validation (supporting); writing – review and editing (supporting). **Gabriela Ibarguchi:** Data curation (supporting); formal analysis (supporting); investigation (supporting); methodology (supporting); validation (supporting); visualization (supporting); writing – original draft (supporting); writing – review and editing (supporting). **Vicki L. Friesen:** Conceptualization (lead); data curation (lead); formal analysis (supporting); funding acquisition (lead); investigation (supporting); methodology (supporting); project administration (lead); resources (lead); supervision (lead); validation (supporting); visualization (supporting); writing – original draft (supporting); writing – review and editing (equal).

## FUNDING INFORMATION

Funding was provided by the *Exxon Valdez* Oil Spill Restoration Office (00169; V.L.F.), a Natural Science and Engineering Research Council (NSERC) Undergraduate Student Research Award (V.F.P.), and an NSERC Discovery Grant (203320; V.L.F). None of our funders had any influence on the content of the submitted or published manuscript. None of our funders required approval of the final manuscript to be published.

## Data Availability

Original data are available as Supplementary Material in the original submission to the Auk. Data will be uploaded to GenBank and Dryad upon acceptance.

## APPENDIX 1 PRIMER DEVELOPMENT

### Microsatellites

A genomic library was developed and screened for dinucleotide (CA) repeats following previously published protocols (Ibarguchi et al., 2000), and PCR primers were developed for two loci (Cco5‐9F: 5′‐TTCCTACCAGTAAAAGAGAGGA‐3′; Cco5‐9R: 5′‐GTACCCCTTTCCTAATTCAAG‐3′; Cco5‐21F: 5′‐TCAAGATGATGAAGACCCTAAT‐3′; Cco5‐21R: 5′‐AGAGTTGCACAGGTTAAATACC‐3′). Six samples were tested for amplification using these primers as well as primers previously developed for microsatellites for Marbled Murrelets (*Brachyramphus marmoratus*, Friesen et al., 2005), Thick‐billed and Common murres (*Uria lomvia* and *Uria aalge*, respectively, Ibarguchi et al., 2000) and Yellow Warblers (*Dendroica petechia*, Dawson et al., 1997). MgCl_2_ concentrations and annealing temperatures were optimized, and PCR products were sequenced to confirm the presence of the expected dinucleotide repeats.

### Introns

Amplifications were attempted on four to six test samples each with 30 pairs of PCR primers previously designed to amplify nuclear introns from vertebrates (Friesen et al., 1997, 1999, unpublished data) using previously published protocols with the addition of 62.5 μg mL^−1^ BSA and 0.01 mg mL^−1^ gelatin. Various annealing temperatures and concentrations of MgCl_2_ and DMSO were tested to optimize amplifications. Two loci for which clean sequence could be derived consistently were then chosen for population screening (Table A1).

**TABLE A1:** PCR primers and annealing temperatures for nuclear loci screened in Pigeon Guillemots.

Locus	Primer sequence (forward/reverse)	Annealing temperature (°C)
Microsatellites		
Cco5‐9^a^	TTCCTACCAGTAAAAGAGAGGA/	55
	GTACCCCTTTCCTAATTCAAG	
Cco5‐21^a^	TCAAGATGATGAAGACCCTAAT/	55
	AGAGTTGCACAGGTTAAATACC	
Dpu16^b^	ACAGCAAGGTCAGAATTAAA/	61
	AACTGTTGTGTCTGAGCCT	
Uaa5‐8^c^	CAGTTTCTTTAAGTCGTGCCAG/	60
	CACTTAGGTCCAAAACCTAACC	
Introns		
Cytochrome *c*	GAAAAAGGAGGCAAGCACAAGACTGG/	53
Intron I (CYTC)^a^	TGTTCCTGGGGATGTACTTCTTTGGATT	
Ribosomal protein 40	GGGCCTGATGTGGTGGATGCTGGC/	63
Intron V (RP40)^d^	GCTTTCTCAGCAGCAGCCTGCTC	

^a^ Present study.

^b^ Dawson et al. (1997).

^c^ Ibarguchi et al. (2000).

^d^ Congdon et al. (2000).

**TABLE A2:** Table of significant linkage disequilibrium for nuclear loci within sampling locations.

Locus	Uaa5‐8	Dpu16	Cco5‐9	Cco5‐21	RP40	CYTC
Andr						
Uaa5‐8						
Dpu16	−					
Cco5‐9	−	−				
Cco‐5‐21	+	−	+			
RP40	−	−	−	−		
CYTC	+	−	−	−	−	
Cook						
Uaa5‐8						
Dpu16	−					
Cco5‐9	−	−				
Cco‐5‐21	−	−	+			
RP40	−	−	−	−		
CYTC	−	−	−	−	−	
PWS						
Uaa5‐8						
Dpu16	−					
Cco5‐9	−	−				
Cco‐5‐21	−	−	−			
RP40	+	−	−	−		
CYTC	−	−	−	−	−	
WVan						
Uaa5‐8						
Dpu16	−					
Cco5‐9	−	−				
Cco‐5‐21	−	−	−			
RP40	+	+	−	−		
CYTC	−	+	−	−	+	
SGeo						
Uaa5‐8						
Dpu16	−					
Cco5‐9	+	−				
Cco‐5‐21	−	−	−			
RP40	−	−	−	−		
CYTC	−	−	−	−	−	
cCal						
Uaa5‐8						
Dpu16	−					
Cco5‐9	−	−				
Cco‐5‐21	−	−	−			
RP40	−	+	−	−		
CYTC	−	+	−	−	+	

*Note*: Significance at *α* = .05 is indicated by +. Location abbreviations are in Table 1.

**TABLE A3:** Estimates of *F*_ST_ for pairwise comparisons of sampling locations of Pigeon Guillemots based on introns (above diagonal) and microsatellites (below diagonal). Location abbreviations are in Table 1.

	Andr	Fox	Shum	Semi	Cook	PWS	SEAK	WVan	SGeo	cOre	cCal
Andr		0.04	0.10	0.06	0.04	0.06*	0.13*	−0.01	0.07*	0.06*	0.26**
Fox	−0.01		−0.03	0.04	0.05	0.03	0.03	0.03	0.05	0.08*	0.29**
Shum	0.01	0.03		−0.01	0.03	−0.01	0.14*	0.02	0.02	0.03	0.17*
Semi	0.02	0.00	0.00		−0.02	−0.03	0.19*	−0.03	−0.02	−0.02	0.04
Cook	0.01	0.01	0.03*	0.02		0.00	0.18**	−0.03	−0.01	−0.01	0.08**
PWS	0.01	−0.01	0.02	0.00	0.00		0.20**	−0.01	0.01	−0.01	0.08*
SEAK	0.10*	0.09*	0.09*	0.07*	0.07*	0.07**		0.16*	0.17**	0.25**	0.46**
WVan	0.12**	0.09*	0.10*	0.03	0.07**	0.07**	0.00		−0.02	−0.03	0.10*
SGeo	0.17**	0.16**	0.11**	0.08**	0.14**	0.15**	0.09**	0.02		0.01	0.07*
cOre	0.13**	0.11**	0.10**	0.08**	0.12**	0.12**	0.05*	0.05*	0.10**		0.05*
cCal	0.28**	0.26**	0.23**	0.20**	0.23**	0.23**	0.17**	0.20**	0.21**	0.04*	

* Significantly different from 0 at *α* = .05 before B–Y correction.

** Significantly different from 0 at *α* = .05 after B–Y correction.

**TABLE A4:** Estimated mean and standard deviation (Stdev) of Ln Pr(*K*), and delta *K* calculated for values of *K* from 1–5 using data from STRUCTURE in STRUCTURE HARVESTER.

*K*	Mean LnP(*K*)	Stdev LnP(*K*)	Δ*K*	Ln Pr(*x*|*K*)
A: admixture, without location as prior information				
1	−2487	0.573	n/a	2.385^−19^
2	−2444	14.76	**4.056**	**1.000**
3	−2461	31.60	2.336	4.266^−8^
4	−2552	76.14	0.664	1.602^−47^
5	−2693	125	1.738	6.565^−109^
B: no admixture, location as prior information				
1	−2487	0.340	n/a	3.527^−93^
2	−2355	1.080	**60.55**	6.379^−36^
3	−2289	1.351	46.64	4.518^−07^
4	−2286	5.950	1.302	1.395^−05^
5	−2274	8.135	n/a	**1.000**

*Note*: All analyses run with correlated allele frequencies for 10 replicates with (A) admixture, without location as prior information, and (B) without admixture, with location as prior information. “n/a” indicates not applicable. The most likely value is in bold.

**TABLE A5:** Type I and Type II error rates and posterior probabilities for each scenario calculated from DIYABC.

True scenario	Type I error rate	Type II error rate	Posterior probability
Scenario 1	0.02	0.026	0.8285 [0.8123,0.8446]
Scenario 2	0.006	0.022	0.0342 [0.0000,0.1101]
Scenario 3	0.04	0.018	0.1373 [0.0562,0.2184]
